# The G protein-coupled receptor-related gene signatures for predicting prognosis and immunotherapy response in bladder urothelial carcinoma

**DOI:** 10.1515/biol-2022-0682

**Published:** 2023-08-10

**Authors:** Zhengqiang Wan, Yinglei Wang, Cheng Li, Dongbing Zheng

**Affiliations:** Department of Thoracic Surgery, The First People’s Hospital of Suining, Suining, Sichuan, China; Department of Urology, Yantai Affiliated Hospital of Binzhou Medical University, Yantai, Shandong, China; Binzhou Medical University, Yantai, China; Yantai Affiliated Hospital of Binzhou Medical University, Yantai, Shandong, China

**Keywords:** bladder urothelial carcinoma, G protein-coupled receptors, tumor microenvironment, immune checkpoint, cellular communication, scRNA, immunotherapy

## Abstract

Bladder urothelial carcinoma (BLCA) is the most common malignant tumor of the urinary tract with a high lethality rate, and its immunotherapy resistance and tumor recurrence have become a major challenge in its clinical treatment. G Protein-Coupled Receptors (GPRs) are the largest family of receptors on the cell membrane surface, involved in multiple signaling pathways, and are excellent targets for oncology drug action. The transcriptome profile, single cell transcriptome profile, and clinical data of BLCA were extracted and integrated from TCGA and GEO databases, respectively. The GPR-related genes were obtained from GSEA-MSigDB database. The GPR-related gene signatures of 15 genes were constructed by using the methods of least absolute shrinkage and selection operator regression, multifactor Cox model. At the same time, tumor microenvironment (TME)-score signatures were constructed based on the immune microenvironment of BLCA, and GPR-TME-score signature was further constructed. The stability of this model was verified by using the external dataset GSE160693. We constructed risk groups by combining BLCA patient prognostic information, and with the help of BLCA scRNA transcriptome profiling, we explored differences in prognosis, immune scores, cell–cell interactions, tumor mutational burden, immune checkpoints, and response to immunotherapy in each risk group. We found that the GPR-TME-score signature was an independent prognostic factor for BLCA patients. the TME-score was a protective factor for the prognosis of BLCA patients. Among BLCA patients, GPR-high + TME-low risk group had the worst prognosis, while GPR-high + TME-high risk group had the best prognosis, and the latter had better immune score and immunotherapy response. The above differences in immune response among the subgroups may be related to the higher immune cell infiltration in the GPR-high + TME-high group. GPR-related gene signatures and TME are closely related to BLCA prognosis and immunotherapy, and GPR-related gene signature can be a useful tool to assess BLCA prognosis and immunotherapy response.

## 1 Introduction

Bladder cancer is one of the most common malignant tumors of the urinary system. 20–30% of patients with bladder cancer have developed muscle-invasive bladder cancer (MIBC) at the time of diagnosis, and 20–25% of non-muscle-invasive bladder cancer can progress to MIBC [1,2]. Nearly 50% of patients with MIBC treated only with radical cystectomy develop metastases to other sites and have a very poor prognosis [3]. The methotrexate, vincristine, gemcitabine, and cisplatin regimen is currently the first-line chemotherapy regimen for bladder cancer, and although patients can be effectively remitted or even cured after conventional postoperative bladder infusion chemotherapy, the recurrence rate is as high as 60% [4]. The malignancy of recurrent bladder tumors will gradually increase and eventually develop into muscle-infiltrating bladder tumors. With the recurrence of tumor, many patients are no longer sensitive to new rounds of chemotherapy, which seriously affects the prognosis of patients [5]. Immunotherapy is based on tumor immunology, which involves several physiopathological processes: (i) Tumor specific antigens must be recognized, presented, and expressed by major histocompatibility complex molecules on the surface of antigen-presenting cells (APCs), which must receive accurate second messenger activation signals (such as B7-1 or B7-2) to achieve an immune phenotype; (ii) Once activated, APCs move to lymphoid tissues, where they interact with T lymphocytes; (iii) If APCs are fully immunocompetent, they induce the generation of CD8 + cytotoxic T lymphocytes (CTL) and natural killer (NK) effector cells that move to the TME and generate anti-tumor immune responses [6]. These effector cells move to the TME and generate an anti-tumor immune response. The discovery of immune checkpoint inhibitors (ICIs) such as programmed cell death factor 1 (PD-1) antibodies, programmed cell death molecule ligand 1 (PD-L1) antibodies, CTL-associated protein 4 (CTLA-4) antibodies, co-stimulatory receptor agonists, photoimmunotherapy, cytokine therapies, and chimeric antigen receptor therapies, has led to the development of a number of new technologies [7]. Therapy, chimeric antigen receptor T (CAR-T) cell therapy, and other therapeutic modalities have provided new directions for the treatment of locally progressive and metastatic bladder cancer [8]. Foreign studies have concluded that upregulation of PD-L1 expression is closely associated with immune evasion in early bladder cancer, and high PD-L1 expression has been shown to be associated with high-grade bladder cancer and worse clinical prognosis [9]. In a single-arm phase II KEYNOTE-052 clinical trial, pembrolizumab was shown to have a durable clinical response in cisplatin-intolerant patients with advanced bladder cancer and was associated with prolonged overall survival, particularly in patients with high PD-L1 expression [10].

G Protein-Coupled Receptor (GPR) is the largest family of membrane receptors linked to G proteins and can be activated by different ligands such as hormones and peptides. Its binding to ligands can activate intracellular G proteins and regulate different physiopathological responses through different signaling pathways. The human genome contains several GPR genes, accounting for about 3% of the total human genome, and GPR has become one of the most important drug targets for drug development [11]. The related research has found that GPR is also closely related to the development of many tumors, and it is involved in the occurrence and development of many tumors such as gastric cancer, colorectal cancer, lung cancer, bladder cancer, and breast cancer, and has the role of promoting tumor growth [12,13]. In addition, GPR is closely related to a variety of malignant biological behaviors such as proliferation, invasion, and metastasis of tumor cells, involving multiple signaling pathways [14]. Zhan et al. [15] found that the GPR signaling pathway promotes the acquisition of drug resistance by tumor cells through the activation of transcription factor Gli downstream of the Hedgehog signaling pathway. However, it is not clear how GPR-related genes affect the prognosis of bladder cancer patients and whether they can predict the response to immunotherapy in bladder cancer patients.

In this study, we used TCGA and GEO databases to screen and identify GPR related gene signatures closely related to the prognosis of bladder cancer, explore their interaction with bladder urothelial carcinoma (BLCA) immune microenvironment, and use this gene signatures to predict the response of BLCA to immunotherapy. In conclusion, our study is the first to explore the impact of GPR related gene signatures on the diagnosis, prediction, and immunotherapy of BLCA, providing reference for personalized immunotherapy of BLCA patients.

## 2 Materials and methods

### 2.1 Data collection and processing

We obtained the original transcriptome data and standardized RNA seq data of 430 BLCA patients and 8 normal bladder tissues from the University of California, Santa Cruz database. The clinical pathological characteristics, follow-up data, and copy number variation (CNV) data of BLCA patients are downloaded from the TCGA-GDC portal. BLCA scRNA transcriptome data (ID: GSM5329919 and GSM5751919) were obtained from the GEO database. Repetitive samples and samples with total survival time of less than 30 days were excluded. In the risk grouping of GPR-score and tumor microenvironment (TME) score, we only used the data of BLCA tumor patients, including transcriptome data and survival data. All data analysis in this study was completed by R language (version 4.0.3). GPR-related genes were obtained from GSEA-MSigDB database. The transcriptome data of 22 immune cells were obtained from CIBERSORT database. The validation dataset is obtained from the GEO database, and its ID is GSE160693.


**Informed consent**: Informed consent has been obtained from all individuals included in this study.
**Ethical approval**: The research related to human use has been complied with all the relevant national regulations, institutional policies, and in accordance with the tenets of the Helsinki Declaration, and has been approved by Ethics Committee of Binzhou Medical University.

### 2.2 Identification of GPR-related genes differentially expressed in BLCA

Differentially expressed genes (DEG) between tumor and normal samples were screened using the R studio “Limma” software package. |Log2FC| > 1, *P* < 0.05, and false discovery rate (FDR) <0.05 were set as cut-off values for DEG in the TCGA cohort. Univariate Cox regression was used to identify prognostic GPR-related genes. Genes with *P* values <0.05 in the Cox regression were identified as prognostic genes for further least absolute shrinkage and selection operator (LASSO) regression analysis.

### 2.3 Tumor immune microenvironment analysis

We downloaded TCGA-BLCA cohort gene expression data and LM22 gene signatures. With the help of the online platform CIBERSORT database, we analyzed the level of BLCA immune microenvironment infiltration, combined with survival data of BLCA patients, and classified them into different risk groups.

### 2.4 Construction and validation of the diagnostic signature

Univariate Cox regression was utilized to evaluate whether the DEGs had an impact on the survival status of TCGA-BLCA cohort based on the DEGs obtained previously, and the lasso Cox regression method (R package “glmnet”) was further used to shrink the candidates to construct the most suitable gene signature, and finally bootstrap model was utilized to further optimize the gene signature model to obtain the GPR-score model. Based on the results of CIBERSORT of TCGA-BLCA cohort, combined with the survival files, we screened the immune cells with good prognosis using the optimal cutoff analysis and constructed a TME-score model using bootstrap. The receiver operating characteristic (ROC) curves were utilized to verify the reliability of the above models.

### 2.5 Constructing risk subgroups

We divided the GPR-score into low- and high-risk groups according to the BLCA patient survival median time and similarly divided the TME score into low and high risk groups. In the combined analysis of GPR score and TME score on the prognosis and immunotherapy of BLCA patients, to remove the influence of confounding factors on the outcome, we divided them into three risk groups: GPR-low + TME-high, GPR-high + TME-low, and mixed (GPR-low + TME-low and GPR-high + TME-high). Prognostic differences between the various risk groups were investigated by Kaplan–Meier (KM) analysis (R package “survival”). In addition, “survminer,” “RMS” and “timeROC” R packages were used to complete ROC analysis.

### 2.6 Independent prognostic analysis of the risk score

We collected the clinical characteristics (Age, T stage, and N stage) of BLCA patients in TCGA cohort and analyzed them together with GPR-TME-score by univariate and multivariate Cox regression to screen independent risk factors for BLCA prognosis.

### 2.7 Calculation of GPR-TME-score for different risk groups tumor mutation load

We downloaded the CNV data of BLCA from TCGA database, and the tumor mutational burden (TMB) was calculated by the formula: TMB (mut/mb) = total number of mutations (including synonymous, non-synonymous point mutations, substitution, insertion, and deletion mutations)/size of target region coding area. With the help of the “oncoplot” software package, the waterfall plot shows the different characteristics of the different risk groups of the GPR-TME-score.

### 2.8 Differences in expression levels of immune checkpoint-related genes among each risk groups

The list of immune checkpoint-associated genes is given in Table S1, and we calculated the expression levels of immune checkpoint-associated genes in each risk group using BLCA transcriptomic data, and the results are presented in box plots, and *P* < 0.05 was considered statistically significant.

### 2.9 Single-cell data analysis of interactions between cells in each risk group

In this study, the BLCA scRNA-Seq datasets were GSM5329919 and GSM5751919, and the R package “Seurat” was used for cell quality control, filtering low-abundance genes (genes expressed in less than three cells) and low-quality cells (number of genes ≤200 or ≥5,000, percentage of mitochondrial genes ≥10%, and percentage of hemoglobin genes ≥5%). The filtered data were normalized for subsequent analysis, and the QC data were subjected to Principal Components Analysis (PCA), t-distributed stochastic neighbor embedding, and uniform manifold approximation and projection (UMAP) for dimensional clustering and cell type annotation using cytomolecular markers, which are given in Table S2. Finally, we used the R package “cellchat” to analyze the interactions between major cell types in each risk group.

### 2.10 Immunotherapy forecast

The half maximal inhibitory concentration (IC_50_) of chemotherapeutic drugs in clusters was calculated using the prophetic algorithm, while we applied the cancer immunome Atlas database to observe the response to immunotherapy in patients with different risk groups and the signaling pathways that were shared by the risk group and ICIs response group were also analyzed using the proteomaps database.

### 2.11 Statistical analysis

All statistical analyses were performed by R4.1.0. Wilcoxon test was used to evaluate the differences in expression levels between BLCA samples and normal tissues as well as each risk group. The relationship between each risk group and neoadjuvant treatment response was assessed by the chi square test.

## 3 Results

### 3.1 Construction and validation of PGR-score model

**Figure 1:**
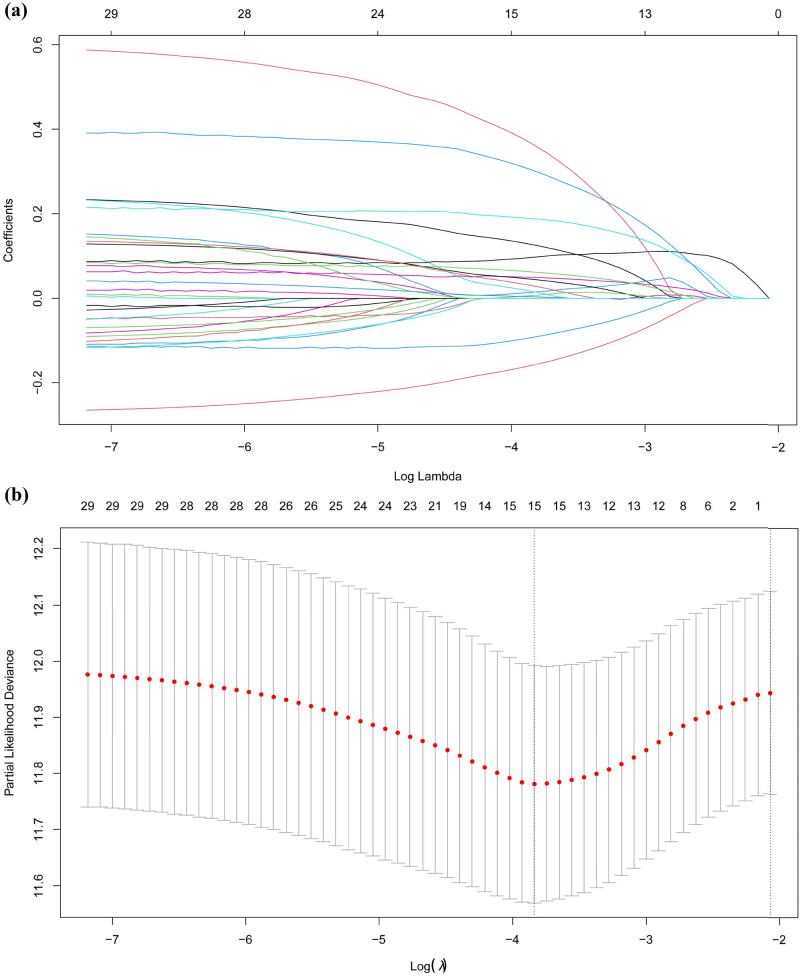

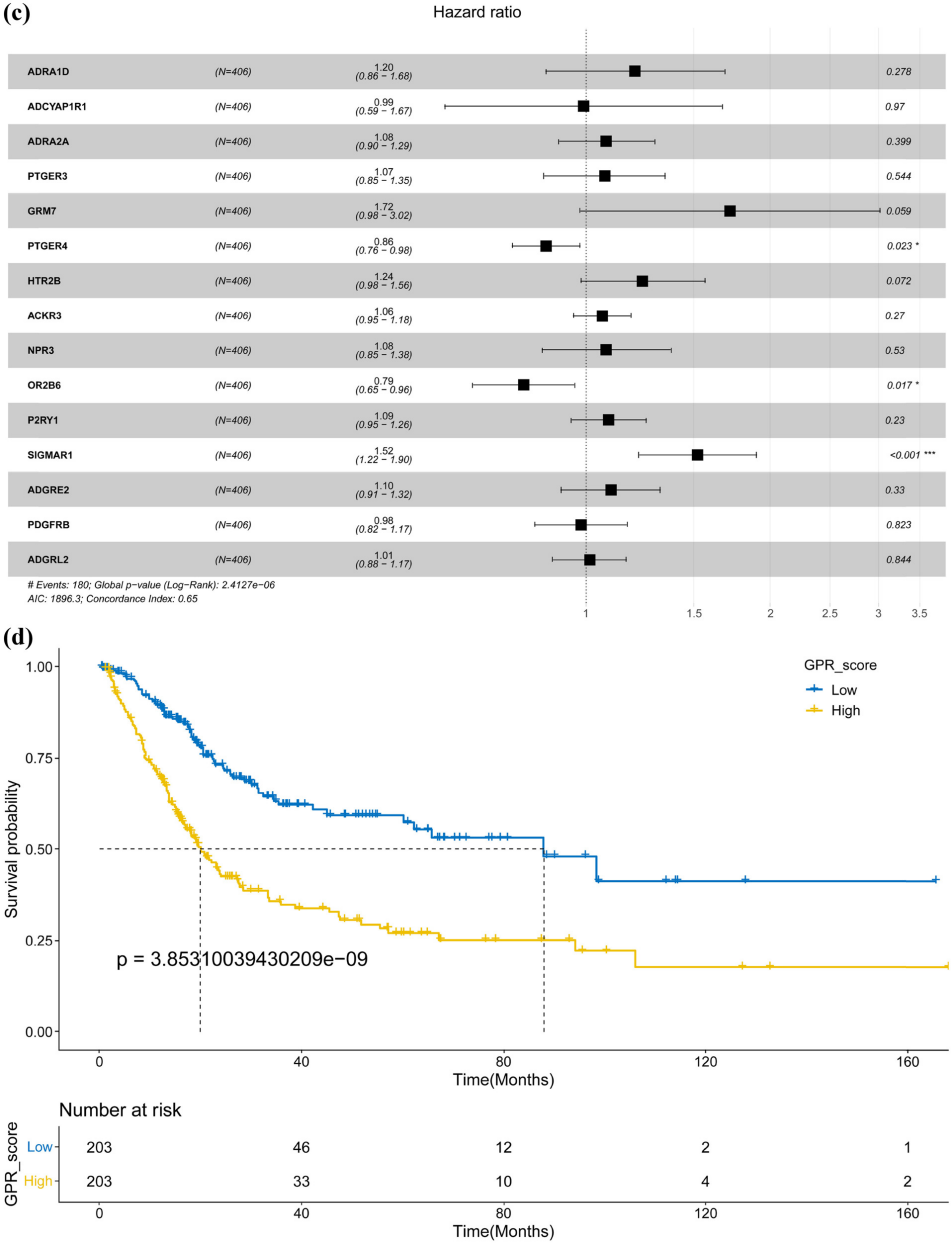
(a) LASSO coefficient pathway plots of GPR gene signatures associated with BLCA prognosis. (b) Cross-validation curves of LASSO regression. (c) Forest plot of multifactor regression analysis of GPR-score gene signatures. (d) Effect of GPR-score high and low risk groups on the prognosis of BLCA patients. *P* < 0.05 was considered statistically significant.

First, we screened prognosis-related GPR-related genes using univariate regression analysis, and a total of 29 prognosis-related genes were obtained, and their results were presented in heat maps (Figure S1). Further, we optimized the variables using LASSO regression and multifactor Cox regression analysis, and screened a total of 15 variables, combined with the median survival of patients, divided the GPR-score into low- and high-risk groups, and constructed the PGR-score model using Bootstrap model (Figure 1a–d). In the multifactorial Cox regression analysis, we found that genes SIGMAR1 (HR = 1.52, *P* < 0.001), OR2B6 (HR = 0.79, *P* = 0.017) and PTGER4 (HR = 0.86, *P* = 0.023) were independent risk factors for the prognosis of BLCA patients, OR2B6 and PTGER4 were protective factors for BLCA patients, while the opposite was true for SIGMAR1 (Figure 1c). Using KM curves to verify the survival difference between GPR-score low and high groups, we found that high GPR-score means a worse prognosis (*N* = 406, *P* = 3.853 × 10^−9^) (Figure 1d).

### 3.2 Construction and validation of the TME-score model

**Figure 2:**
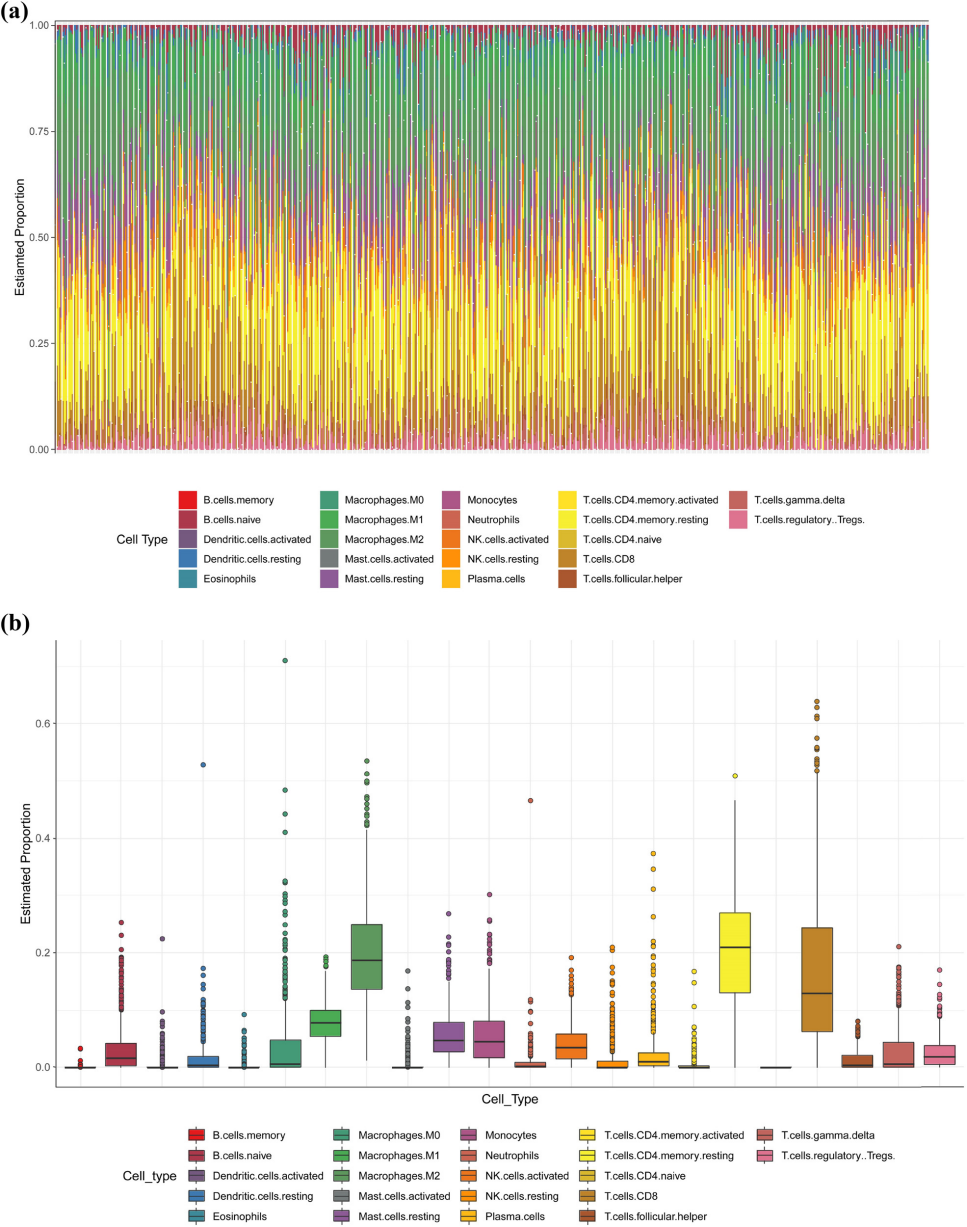

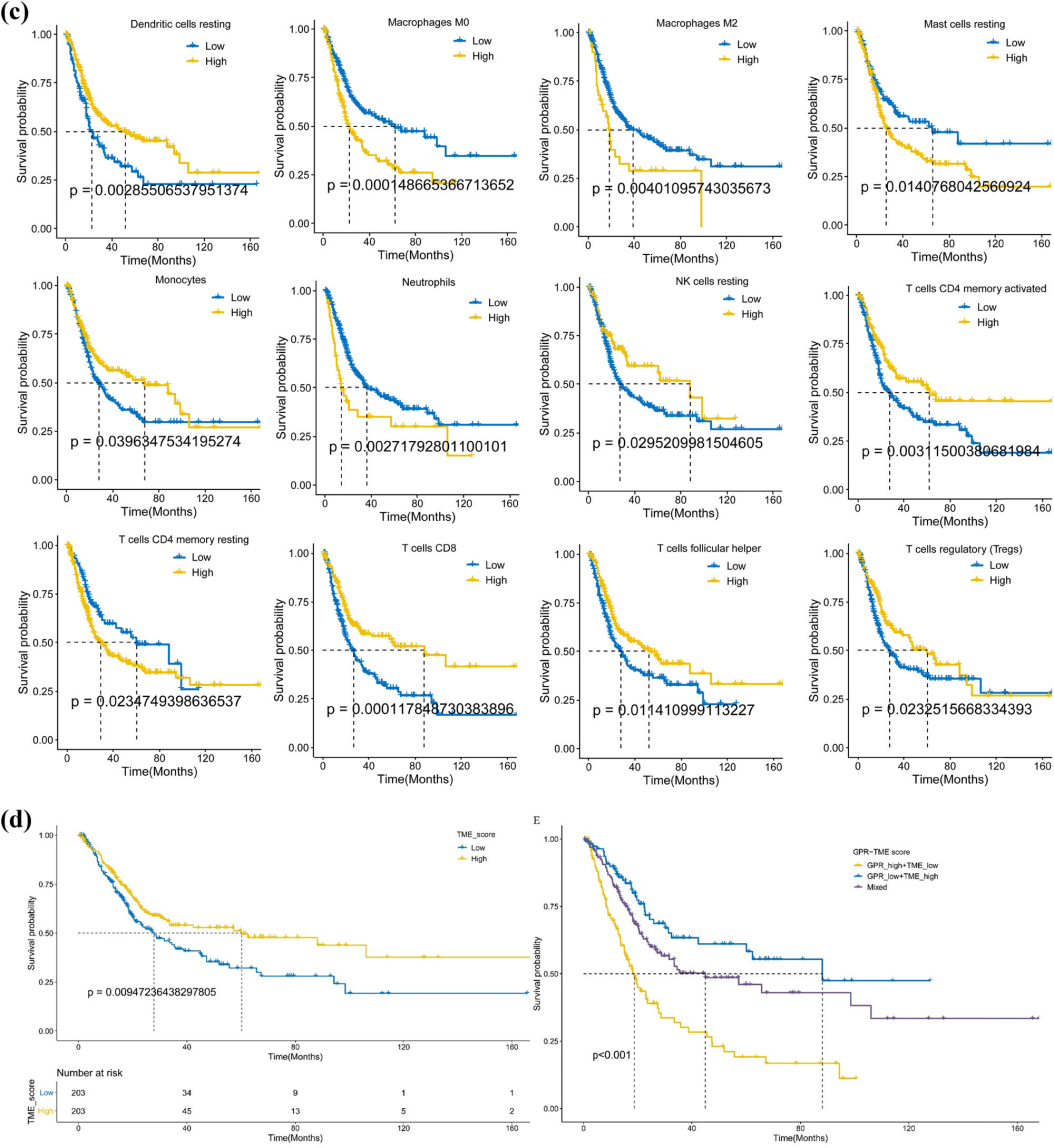
(a) Immune landscape map of BLCA based on CIBSORT database. (b) Box plot of the number of immune cells based on CIBSORT database. (c) The effect of 22 immune cell subtypes in high-risk and low-risk groups on the prognosis of patients with BLCA based on CIBSORT database. (d) Effect of TME-score high and low risk groups on the prognosis of BLCA patients. *P* < 0.05 was considered statistically significant. (e) Effect of different risk groups of GPR-TME-score on the prognosis of BLCA patients.

We used TCGA-BLCA transcriptome data, and with the help of CIBERSORT database, we analyzed the level of immune infiltration in BLCA. The most abundant immune cell type in the sample was T cells, and CD8 T cells were the most abundant immune cell subtype (Figure 2a and b). KM curves were used to screen immune cell types relevant to the prognosis of BLCA patients, and the TME-score model was further constructed using Bootstrap. We found that a total of 12 immune cell subtypes were associated with the prognosis of BLCA patients (Figure 2c). Current tumor immunology studies suggest that CD8^+^ T cells, CD4 memory activated, and Dendritic cells activated play a protective role in tumors, and the higher their infiltration levels, the better the patient prognosis. Therefore, based on these views, we selected the above three immune cells to construct the TME-score model. Based on the survival data of patients, we divided TME score into TME score low and high risk groups. KM curve indicates that the prognosis of patients in the high TME-score group is better than that in the low group, indicating that TME-score is a protective factor for the prognosis of patients with BLCA (Figure 2d). Finally, to analyze the effects of GPR gene signature and TME on the prognosis of BLCA patients, we further constructed a GPR-TME risk set and validated its diagnostic efficacy with KM curves. we found that patients in the GPR-low + TME-high group had the best prognosis and those in the GPR-high + TME-low group had the worst prognosis (Figure 2e).

### 3.3 Identification of independent risk factors for prognosis of BLCA patients

**Figure 3:**
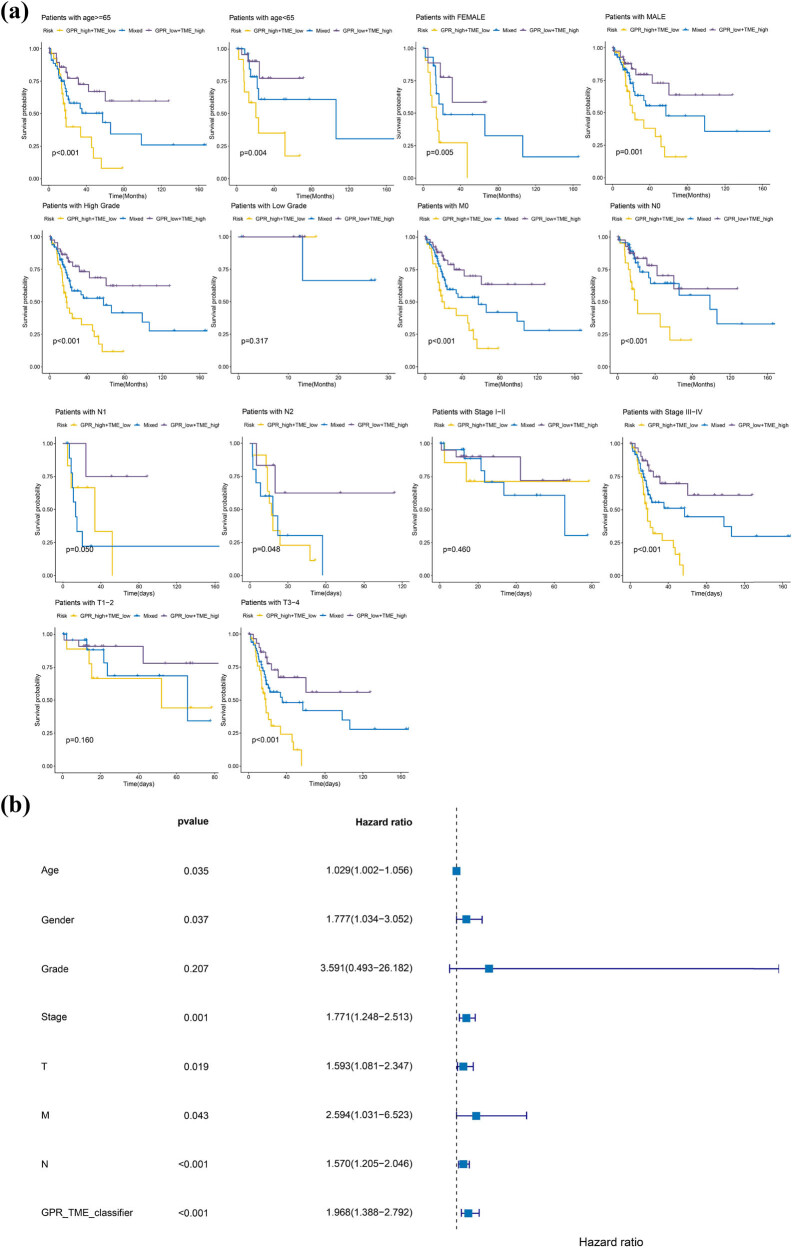
(a) The impact of clinicopathological characteristics of each risk group of GPR-TME-score on the prognosis of BLCA patients. (b) Forest plot of independent risk factors for prognosis in BLCA patients. *P* < 0.05 was considered statistically significant.

Using transcriptomic data from the TCGA-BLCA cohort, we combined patient clinicopathological data (age, gender, differentiation grade, clinical stage, T-stage, N-stage, and M-stage), prognostic information, and GPR-TME-score to identify independent risk factors for prognosis of BLCA patients. From the KM curves of each clinical subgroup, we found that three risk groups were associated with the prognosis of BLCA patients in different clinical subgroups, including age, gender, differentiation grade, clinical stage, T stage, N stage, and M stage (*P* < 0.05), but not statistically significant in differentiated grade clinical subgroups (*P* > 0.05). Among the clinical subgroups, BLCA survival had the best overall prognosis in the GPR-low + TME-high group and the worst prognosis in the GPR-high + TME-low group (Figure 3a). In multifactorial Cox regression analysis, we found that gender (HR = 1.777, *P*-value = 0.037), clinical stage (HR = 1.771, *P*-value = 0.001), T stage (HR = 1.593, *P*-value = 0.019), N stage (HR = 1.570, *P*-value < 0.001), M stage (HR = 2.594, *P*-value = 0.043) and GPR-TME-score (HR = 1.968, *P*-value < 0.01) as independent risk factors for the prognosis of BLCA patients (Figure 3b).

### 3.4 Single-cell transcriptome reveals interactions among risk group cells

**Figure 4:**
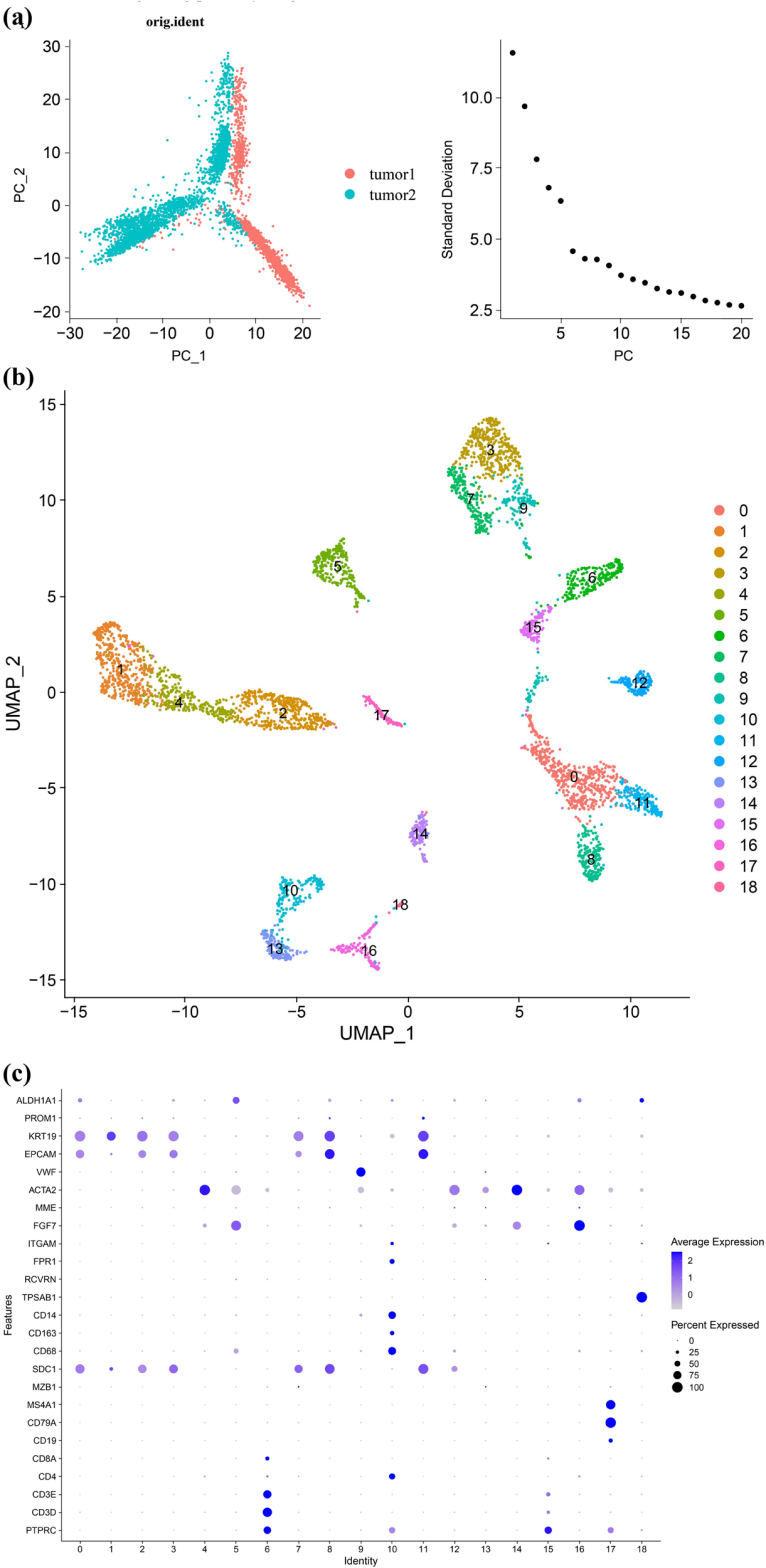

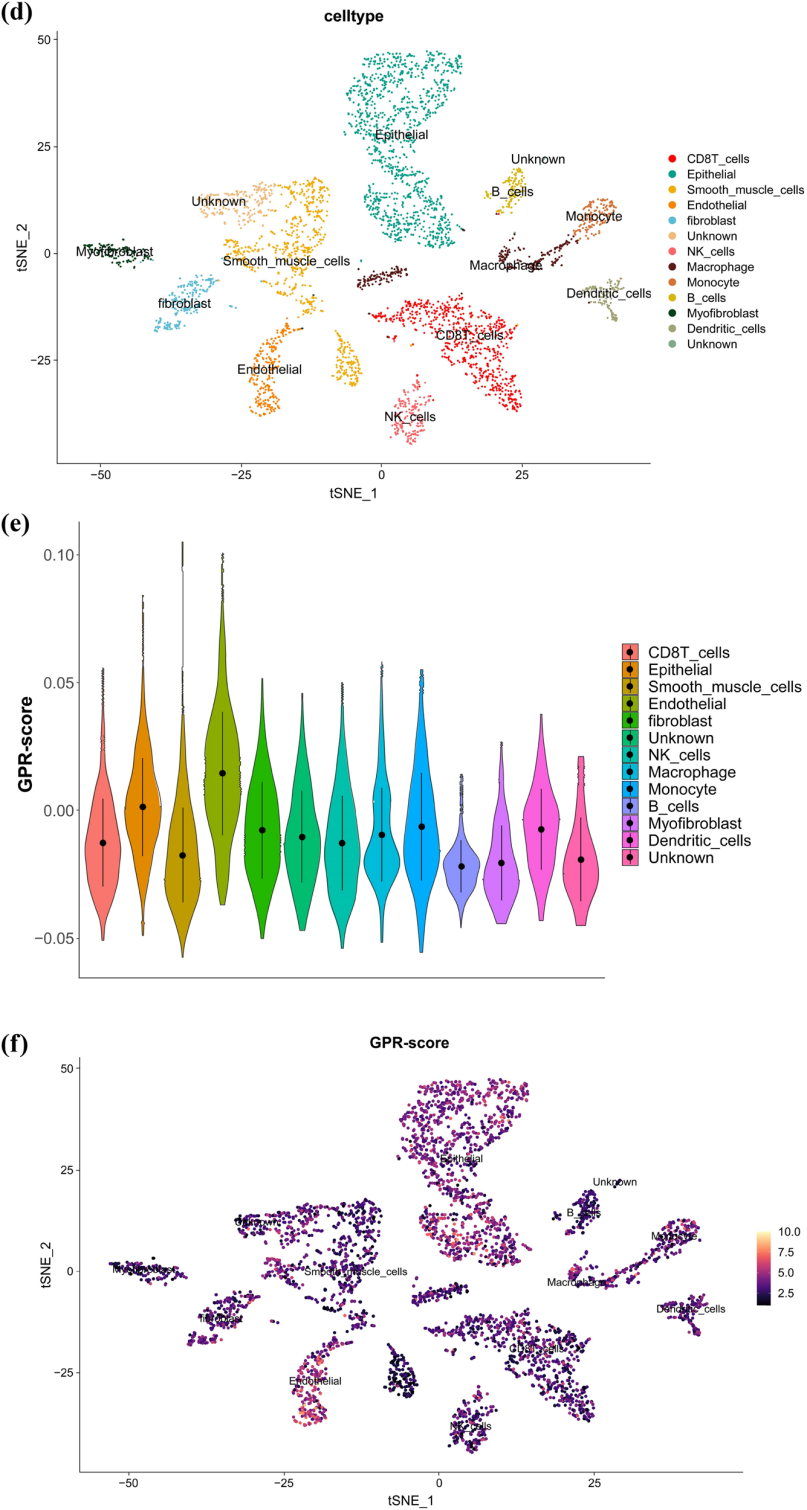

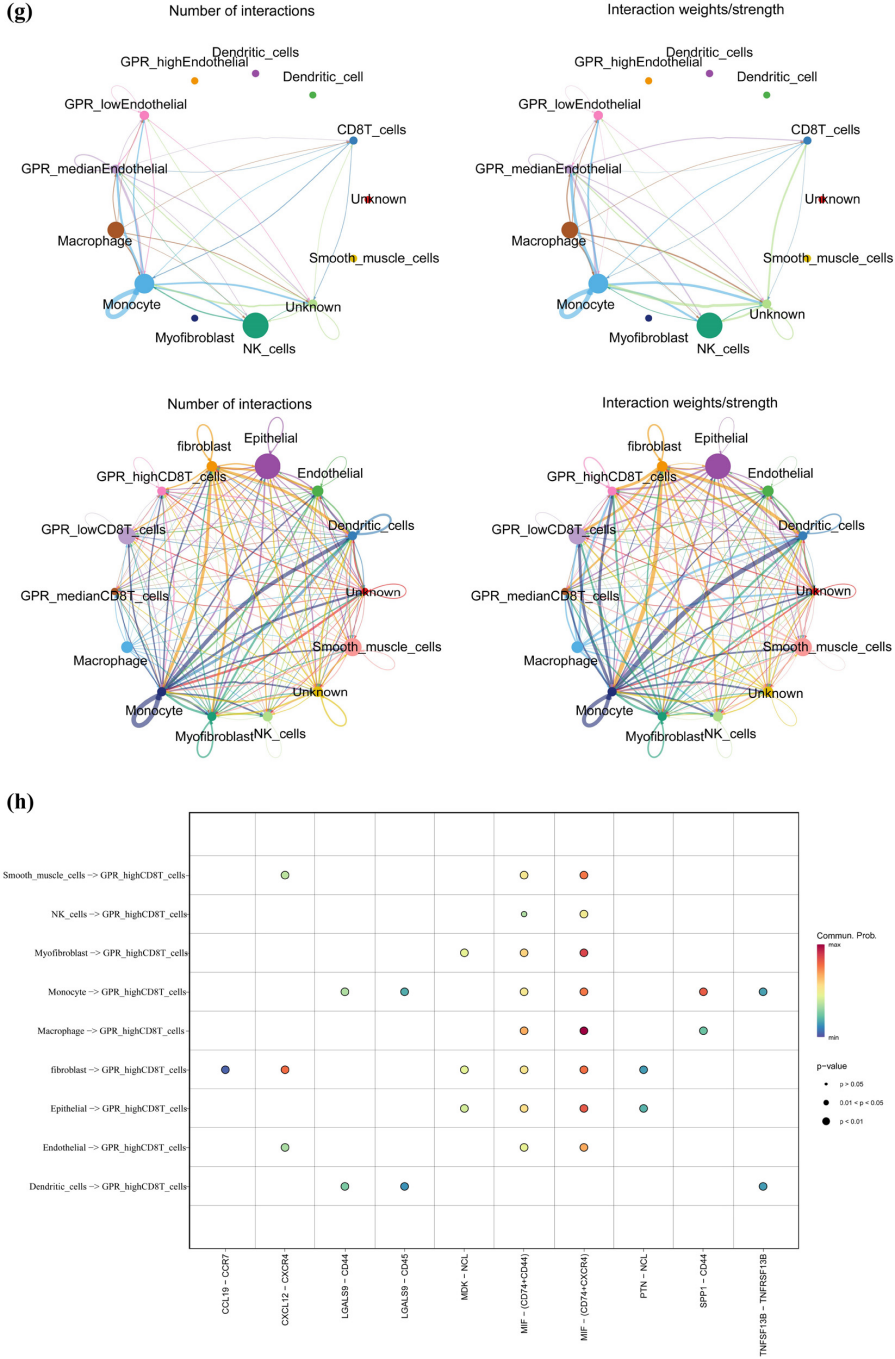

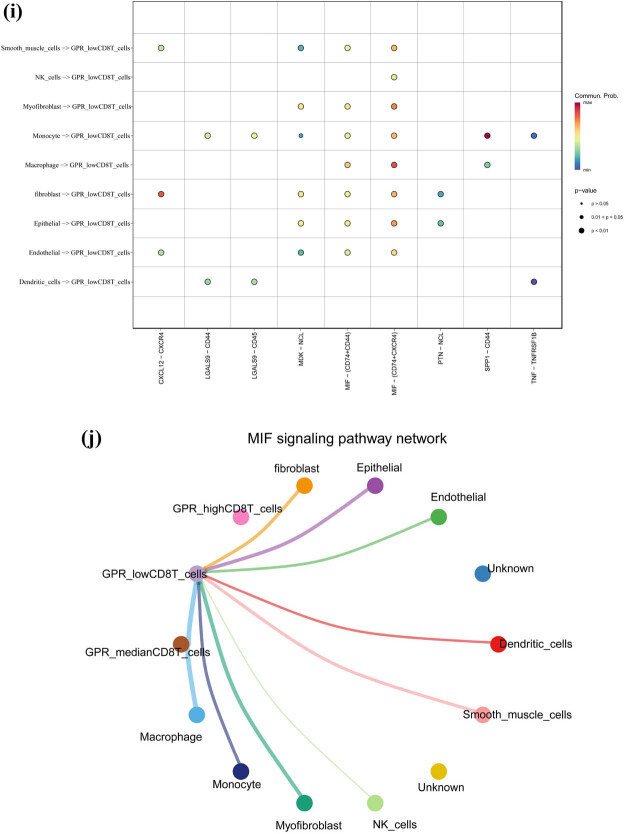
(a) Scatter plot of PCA of BLCA single-cell transcriptome data. (b) Scatter plot of UMAP downscaling clustering of BLCA single cell transcriptome data. (c) Scatter plot of cellular annotation of 18 cell clusters of BLCA. (d) Cellular annotation of BLCA based on UMAP downscaling clustering. (e and f) GPR-score expression levels in each cell type of BLCA. (g) Number and intensity of interactions between endothelial cells and CD8^+^ T cells with other cells in each risk group of GPR-score. (h) Ligand–receptor pair bubble plots of CD8^+^ T cell–cell interactions in GPR-score high-risk groups. (i) Ligand–receptor pair bubble plots of CD8^+^ T-cell interactions in the GPR-score low risk group. (j) Interactions between GPR-score risk groups and other cell types via MIF-signaling-pathway. *P* < 0.05 was considered statistically significant.

Two BLCA samples with transcriptome data from GEO data with sample numbers GSM5329919 and GSM5751919 were used in this study. First, we used the R package “Seurat” to QC the BLCA single cell transcriptome data, filtered out null cells, mitochondrial genes, ribosomal genes, and hemoglobin genes (Figure S2), removed sample differences using PCA, and normalized the data for subsequent analysis. We used the FindVariableSignature function of the R package “Seurat” to find the high-variable genes in the samples (Figure S3), and then performed UMAP downscaling clustering of the high-variable genes to obtain a total of 18 cell clusters by UMAP clustering (Figure 4a and b). The 18 cell clusters were annotated using cell markers (Figure 4c), and the detailed list of cell type markers is shown in Table S2. 13 cell types were obtained, of which 2 cell types could not be clearly classified as a certain cell type, and the largest number of cells were epithelial cells (Figure 4d). Among the 13 cell types, GPR-score is highly expressed in CD8 + T cells of immune cells and in endothelial cells of non-immune cells, suggesting that GPR-score acts in BLCA mainly by affecting endothelial cells and CD8^+^ T cells (Figure 4e and f). Next we further explored the intercellular interactions between CD8^+^ T cells and endothelial cells according to the GPR-score risk score, which was divided into three risk groups: high, median, and low. We found that endothelial cells were not active in intercellular interactions, interacting mainly with NK cells, macrophages, and monocytes, with the greatest intensity of action with macrophages. The CD8^+^ T cells were extremely active in intercellular interactions, both in terms of the number and intensity of cells interacting, with the greatest intensity of action being displayed by epithelial cells (Figure 4g). Given that the CD8^+^ T cells are extremely active in BLCA cell communication, suggesting an important role in intercellular interactions, we further explored the ligand receptors in cell communication of CD8^+^ T cells according to the GPR risk score, and we found that CD8^+^ T cells in both GPR-high and GPR-low risk groups interacting with MIF-(CD74 + CXCR4) ligand–receptor relationships were extremely correlated (Figure 4h and i). Since MIF-(CD74 + CXCR4) ligand–receptors were extremely associated with both GPR-high and GPR-low risk groups, we further explored the MIF-signaling-pathway-network and found that the interaction between CD8 + T cells of different risk groups of GPR-score and other cell types is related to MIF signaling pathway (Figure 4j).

### 3.5 TMB of different risk groups of GPR-TME-score and its ability to predict immunotherapy response

**Figure 5:**
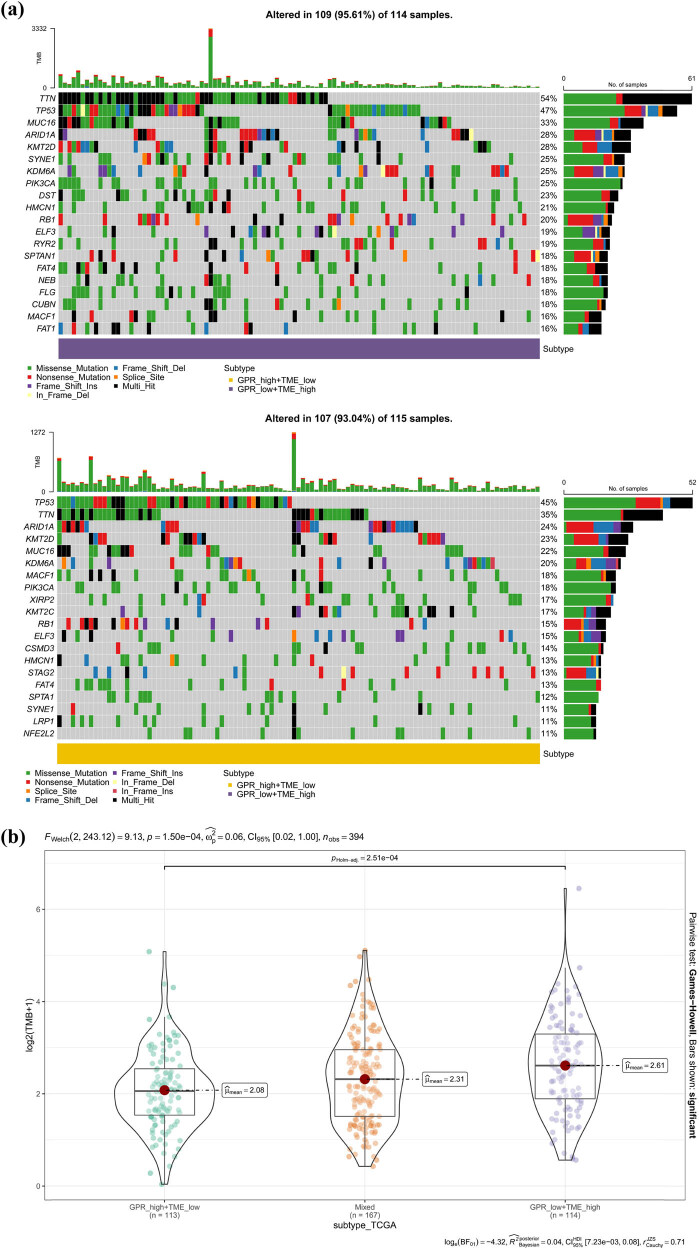

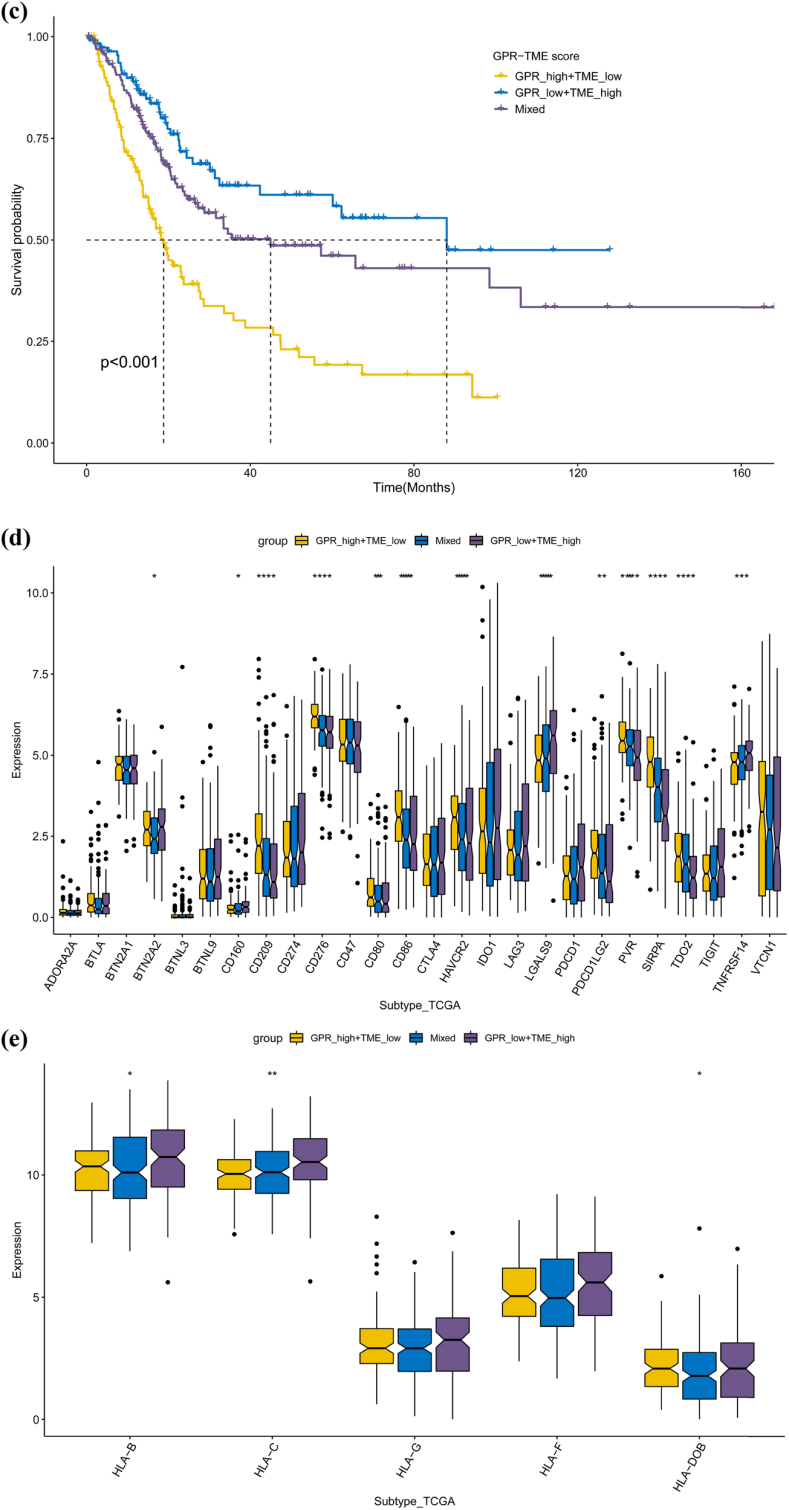

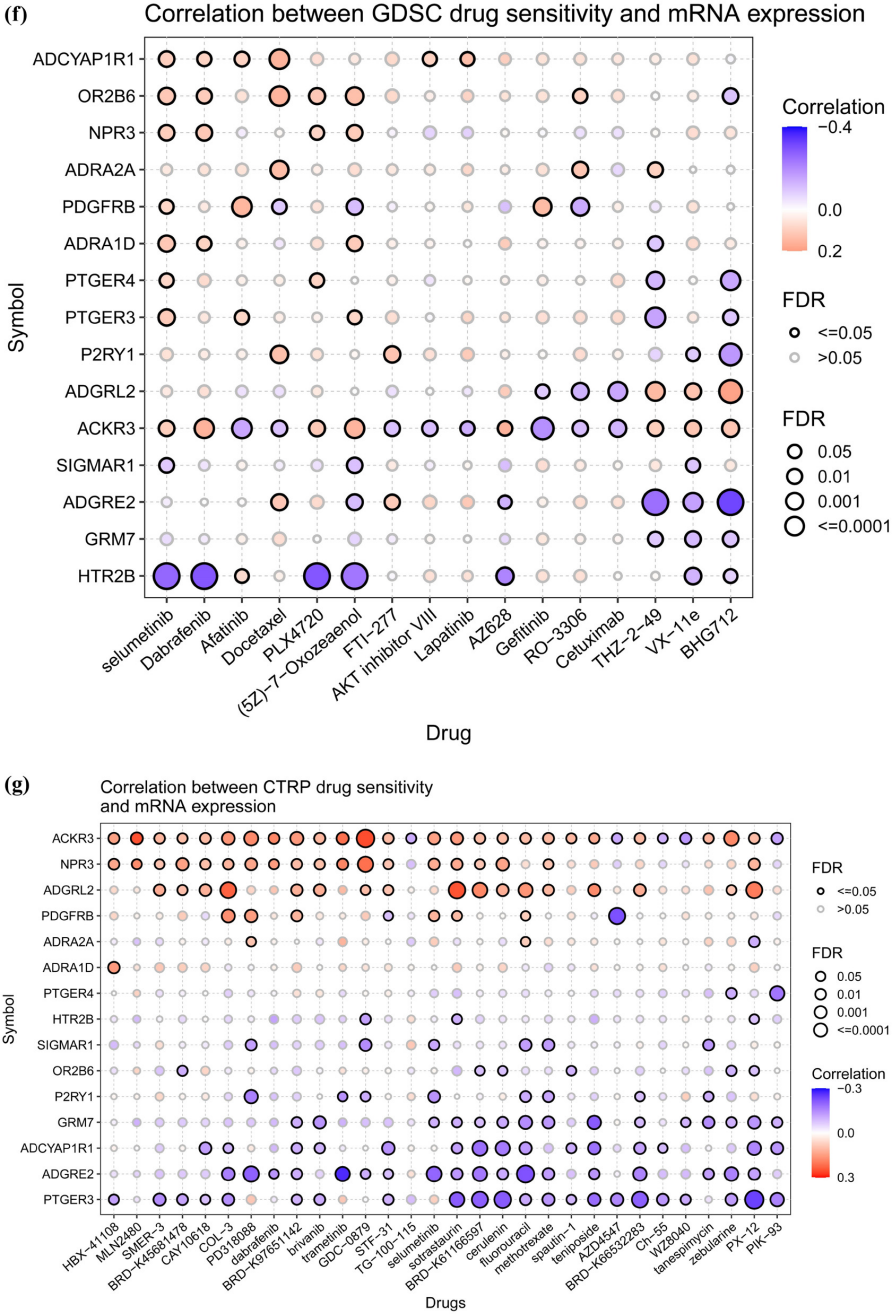

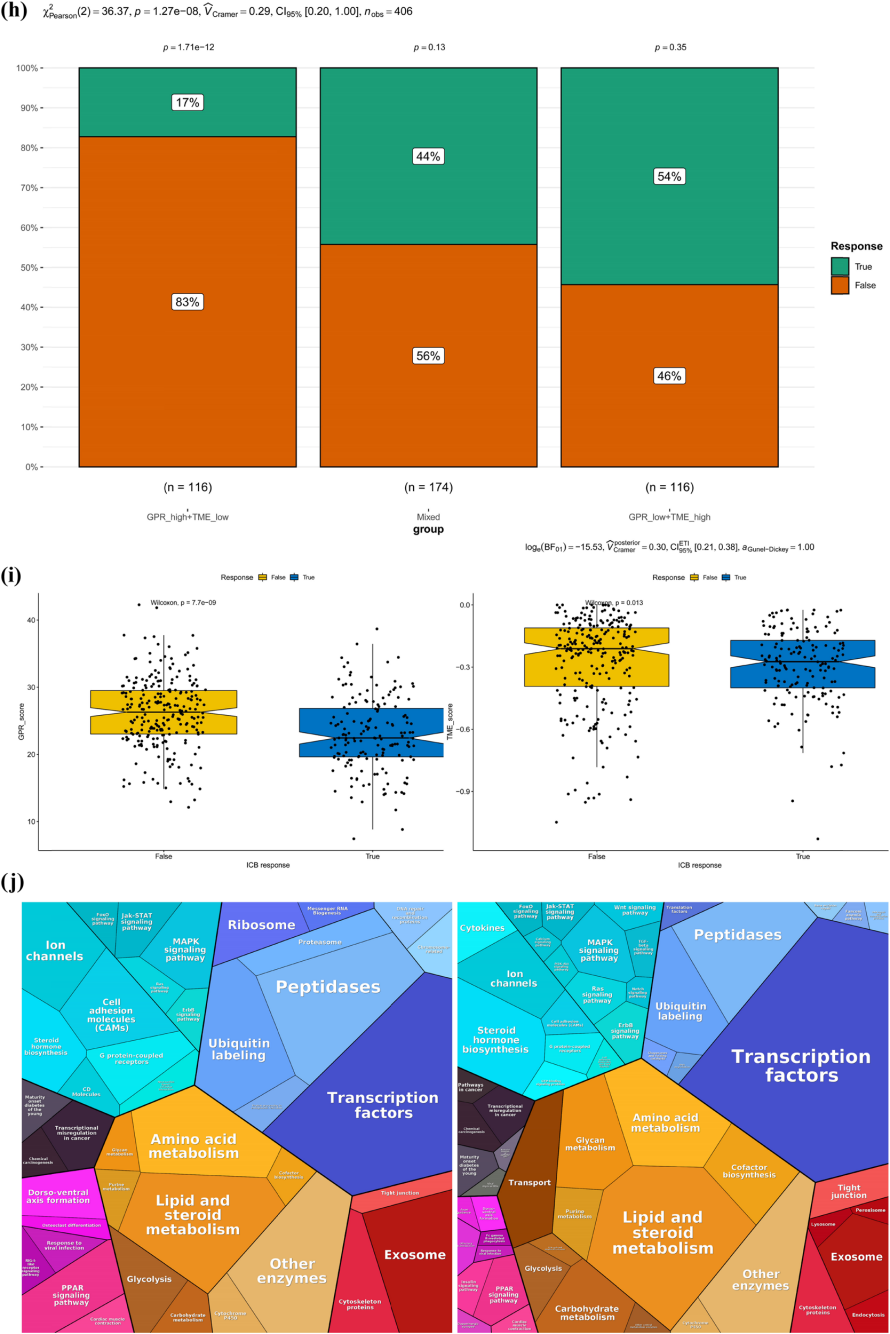
(a) Mutation waterfall plots of high and low GPR-TME-score risk groups. (b) Differences in TMB levels in different risk groups of GPR-TME-score. (c) Effect of TMB in different risk groups of GPR-TME-score on the overall survival rate of BLCA patients. (d) Immune checkpoint-associated gene signatures were differentially expressed in the GPR-TME-score across risk groups. (e) HLA-related gene signatures were differentially expressed in the GPR-TME-score across risk groups. (f) Bubble plot of the correlation between GPR-score gene and targeted drugs based on GDSC database. (g) Bubble plot of the correlation between GPR-score gene signatures and targeted drugs based on CTRP database. (h) Differences in response to immunotherapy among risk groups of GPR-TME-score based on the TIDE database. (i) Box line plot of differential response to ICIs in patients with GPR-score and TME-score. (j) Fragmentation plots of signaling pathways for immunotherapy responders in the GPR-TME-score and their ICI responders acting together based on the proteomap database. *P* < 0.05 was considered statistically significant.

To investigate whether there is a difference in TMB among different risk groups of GPR-TME-score, we explored the level of tumor mutation load in each of their risk groups with the help of CNV data from the TCGA-BLCA cohort to predict the response of different risk groups to immunotherapy. We first depicted the mutational landscape of each risk group and further calculated its TMB. We found that missense mutations were the most frequent mutation type in BLCA, with a lower mutation rate in the GPR-high + TME-low group (93.04%) than in the GPR-low + TME-high group (95.61%), and further calculated the TMB of each risk group and found that the GPR-high + TME-low group had a lower TMB than the GPR-low + TME-high group (95.61%). GPR-high + TME-low group having lower TMB than the GPR-low + TME-high group (*N* = 394, *P* = 2.5 × 10^−4^) suggested that the GPR-low + TME-high group had a better response to immunotherapy (Figure 5a and b). We also calculated the effect of TMB on the prognosis of BLCA patients in each risk group, and found that the GPR-low + TME-high group had the best prognosis and the GPR-high + TME-low group had the worst prognosis (*P* < 0.001) (Figure 5c). To investigate whether there were differences in immune checkpoint expression levels among risk groups, we calculated the expression levels of immune checkpoint-associated genes in the three risk groups. We found that the immune checkpoint-related genes CD209, CD276, CD86, and PDCD1LG2 differed among the risk groups (*P* < 0.05), and we also found that the expression levels of immune checkpoint-related genes in the GPR-low + TME-high group were generally higher than those in the remaining two risk groups, suggesting that the GPR-low + TME-high group had a better response to ICIs (Figure 5d). Human leukocyte antigen (HLA), an independent factor in tumor-associated antigen presentation, plays an important role in antitumor immune response and de novo tumor progression. Here we explored the expression levels of HLA-related genes in each risk group, and found that HLA-related genes HLA-B, HLA-C, and HLA-DOB were differentially expressed in different risk groups (*P* < 0.05), and we also found that the expression levels of HLA-related genes in the GPR-low + TME-high group were generally higher than those in other risk groups (Figure 5e). We then investigated the correlation between GPR-score gene expression levels and chemotherapy drug sensitivity with the GDSC database and CTRP database, and found that their expression levels were very closely related to MEK1/2 locus-targeting drug selumetinib and CTRP drug Cerulenin (Figure 5f and g). Finally, we explored the responsiveness of each risk group to immunotherapy using the TCGA-BLCA cohort with the help of the Tumor Immune Dysfunction and Exclusion Database (TIDE). We found that 17% of the 160 patients in the GPR-high + TME-low group responded to immunotherapy and 83% did not (*P* = 1.71 × 10^−12^), and the immune response in the remaining two risk groups was not statistically significant (Figure 5h). We further analyzed the difference in response levels to immune checkpoint blockade (ICIs) between patients with GPR-score and TME-score, and found that patients with GPR-score (*P* = 7.7 × 10^−9^) and TME-score (*P* = 0.013) responded to ICIS and there was a difference (Figure 5i). Finally, we used transcriptomic data from ICIs-responding patients to screen for differential genes with the help of the R package “Limma,” and used the proteomap database to explore the signaling pathways common to patients in the GPR-high + TME-low risk group responding to immunotherapy and their ICIs-responding counterparts. We found many signaling pathways common to both, such as the P13-AKT-signaling-pathway and MAPK-signal-pathway, suggesting that the GPR-TME-score may influence the response to immunotherapy in BLCA patients through the above pathways (Figure 5j). In conclusion, the GPR-TME-score model can better predict the response to immunotherapy in BLCA patients.

## 4 Discussion

BLCA, one of the most common malignancies of the urological tract, is highly susceptible to drug resistance, and there is a lack of effective therapeutic targets and accurate efficacy prediction models. In this study, we constructed a 15-gene risk signatures in the TCGA-BLCA cohort by univariate Cox analysis and LASSO regression analysis, and a TME signature based on the TCGA-BLCA immune microenvironment, and because the immune microenvironment affects the tumor response to immunotherapy, we combined the above two risk profiles to construct a GPR-TME signatures, and explored the value of this model in BLCA overall survival and immunotherapy. We found that the GPR gene signatures was a risk factor for overall survival in BLCA, while the TME risk signatures was a protective factor for overall survival in BLCA, and the GPR-TME signature was an independent risk factor for overall survival in BLCA. In exploring the level of immune infiltration in BLCA, we found that T cells were the predominant immune cell type in BLCA, and that the level of immune infiltration differed among risk groups, with lower GPR signatures or higher TME signatures implying higher levels of immune infiltration. In BLCA, higher levels of immune infiltration represent a better response to immunotherapy. In exploring the independent risk factors for overall survival in BLCA, we found that gender, clinical stage, T stage, N stage, M stage, and GPR-TME signatures were independent risk factors for their overall survival. In exploring the single cell transcriptome data of BLCA, we found that epithelial cells were the cell type with the highest cell number in BLCA and CD8^+^ T cells were the immune cells with the highest cell number in it. Based on these two cell types, we further explored their intercellular interactions and found that CD8^+^ T cells were extremely active in intercellular interactions, while epithelial cells were far less active than CD8^+^ T cells. Furthermore, the MIF-(CD74 + CXCR4) ligand–receptor is extremely important for CD8^+^ T cells to play intercellular roles. Finally, we investigated whether the GPR-TME signatures could predict the response to immunotherapy in BLCA patients, and the response to immunotherapy differed among risk groups in this model, with lower GPR signatures or higher TME signatures implying better levels of immune response. We applied the model to the ICIs treatment cohort and found that the model could be used to predict the efficacy of ICIs treatment in BLCA patients.

The mechanisms of tumor drug resistance are complex, but most of them are related to epigenetic modifications and mutations in oncogenes, and drug resistance in bladder cancer can be considered as a result of relevant genetic variants [16]. In addition, abnormal non-coding RNA function, generation of bladder cancer stem cells, increased drug efflux, and autophagy are also important causes of drug resistance in bladder cancer [17,18]. Gene expression and regulation play an important role in tumor cell growth and differentiation, invasion, metastasis, and drug resistance. The epithelial-mesenchymal transition (EMT) plays an important role in tumor invasion, metastasis, formation of cancer initiating cells (CICs), and drug resistance. The CXCL5 gene may induce bladder cancer recurrence by activating NF-kB pathway-regulated EMT that causes chemoresistance in bladder cancer cells [19,20]. Li et al. found that FAT10 was highly expressed in bladder cancer cell lines and promoted the formation of EMT and CICs in bladder cancer UMUC-3 cells, leading to cisplatin resistance [21]. Tumor stem cells (CSCs) are defined as a subpopulation of self-renewing and self-protective cancer cells that can differentiate into morphologically and functionally diverse cancer cells with a limited lifespan and are a major factor in tumor drug-resistant recurrence [22]. The production of BCSCs and bladder cancer resistance and recurrence may involve alterations in multiple signaling pathways, such as the KMT1A-GATA3-STAT3 pathway, Hedgehog pathway, Notch pathway, Wnt pathway, JAK-STAT pathway, COX2/PGE2 pathway, YAP1 pathway, and miR34a/GOLPH3 pathway, and may play a key role in maintaining the properties of BCSCs, thus promoting bladder cancer drug resistance and recurrence [23,24,25]. In the gene enrichment analysis of the most relevant signature module genes obtained from WGCNA analysis for each risk group and differential genes in ICIs-responding patients, we found that they were enriched in signaling pathways such as NF-KB signaling pathway, JAK-STAT pathway, and Wnt pathway, which suggested that the above signaling pathways might be related to bladder cancer drug resistance and recurrence.

GPR is a class of transmembrane protein receptors with seven transmembrane helices, which can recognize and bind to various extracellular signaling molecules and undergo structural changes, activating a series of intracellular signaling pathways and ultimately causing changes in cellular state [26]. It is an important target for drug development. In the treatment of tumors, 21 anti-cancer drugs targeting GPR have been approved for marketing [27]. These include the gonadotropin-releasing hormone receptor antagonist Degarelix for advanced prostate cancer and the SMO receptor inhibitors Sonidegib and Vemurafenib for basal cell carcinoma [28]. There are also 23 GPR drugs under development for the treatment of cancer, seven of which target novel targets, including chemokine receptors and proteins in the Wnt signaling pathway [29]. Numerous studies have confirmed that members of the GPR family are closely associated with the development of tumors.

The results of a study suggest that CD97 overexpression increases migration and induces early tumor growth in colon cancer cells [30]. In a mouse model of gastric cancer, CD97 was also shown to promote local tumor growth and metastatic spread [31]. It has been shown that BAI1 can inhibit glioblastoma angiogenesis [32]. In addition, BAI1 has been found to inhibit neovascularization in colon cancer, breast cancer, gastric cancer, and renal cell carcinoma [33]. A study that included 37 patients with pancreatic cancer showed that CD97 was associated with aggressiveness and overall survival of pancreatic cancer [34]. In two independent cohort studies that included 187 and 539 patients with glioblastoma, respectively, CD97 expression was upregulated in stage IV glioblastoma and correlated with overall survival [35]. In univariate Cox analysis of GPR-related genes, we also found that GPR family members were associated with overall survival in BLCA patients, and GPR family-related genes SIGMAR1, OR2B6, and PTGER4 were independent risk factors for overall survival in BLCA patients. In predicting ICIs response, GPR gene signatures could predict the response to ICIs in BLCA patients.

TME is mainly composed of tumor cells, immune cells, stromal cells, endothelial cells and tumor-associated fibroblasts [36]. Several studies have shown that [37,38,39] tumor-associated macrophages (TAMs) and tumor-infiltrating lymphocytes (TILs) are important components of immune cells in TME and are strongly associated with bladder cancer progression, overall clinical survival, and response to immunotherapy. TAMs are important components of immune cells in bladder cancer TME and are characterized by M1-type macrophages and M2-type macrophages. M1-macrophages exert anti-tumor effects by promoting increased expression and release of cytokines such as IL-1β, TNF-α, IL-6, and IL-12 and elevating the helper T cell 1 (Th1)-mediated immune response. M2-macrophages exert immunosuppressive functions by promoting the expression and secretion of immunosuppressive cytokines such as TGF-β 1 and IL-10, thereby promoting tumor progression and metastasis [40]. The study showed that M2-macrophage cells have a strong immunosuppressive function by promoting the expression and secretion of immunosuppressive cytokines such as TGF-1 and IL-10. Related studies have shown that an increased proportion of M2-macrophage infiltration is associated with poorer overall survival in bladder cancer patients [41]. Pfannstiel et al. [42] conducted a study showing that high levels of TIL in bladder cancer were associated with increased expression of immune checkpoints such as PD-1 and PD-L1, tumor antigens, and mutational load, suggesting that bladder cancer patients with high infiltrating lymphocytes would benefit more from ICIs. Initial CD8^+^ T cells are activated by specific antigen stimulation and subsequently differentiate into cytotoxic T cells (CTL) with the assistance of effector Th cells, which can exert cytotoxic killing effects in direct contact with target cells. B lymphocytes have a dual effect of promoting or inhibiting tumor growth in the TME [43]. In an early study carried out by De Visser et al. [44] using transgenic mouse models showed that B cells can promote chronic inflammation and thus promote tumorigenesis and progression. However, some reports [45,46] show that B cells can inhibit the development of other types of tumors and that the higher the number of tumor-infiltrating B cells, the better the overall survival rate. This is because they may exert their antitumor effects through synergistic effects with T lymphocytes or by their own action. Although B cells in the TME may play an important role in inhibiting tumor progression [47,48,49], their effects on bladder cancer are unknown. Although PD-L1 and TMB have been used clinically as biomarkers for immunotherapy selection in many patients, there are still many patients who cannot benefit based on these two markers [50]. By analyzing the tumor immune microenvironment, the response of bladder cancer patients to immunotherapy can be effectively predicted, and how to further modulate TME and improve the anti-tumor capacity of T cells in bladder cancer is a potential direction for future bladder cancer immunotherapy. In this study, with the help of the CIBERSORT database, we explored the infiltration levels of 22 immune cell types in BLCA, TAMs and TILs were its main immune cell types, 12 of which were immune cell subtypes associated with overall survival of BLCA patients, M2-macrophages were risk factors for overall survival of BLCA patients, and CD8^+^ T cells were the protective factors, which is consistent with numerous researchers. Further analyzing the level of immune cell infiltration in each risk group, we found differences in the levels of TAM and TIL infiltration. In the analysis of BLCA single-cell data, we found that CD8^+^ T cells were its main immune cell type, and its transcriptome data also indicated that CD8^+^ T cells were the main immune cell type, and CD8^+^ T cells tended to imply better overall survival, and the KM curves of BLCA confirmed this view. In conclusion, the TME signatures we constructed were able to predict the overall survival and immunotherapy response of patients. A high TME signature implies better overall survival and immune response.

The GPR-TME risk model constructed in this study performed well in predicting the overall survival and immunotherapy response in BLCA patients, but had some shortcomings. First, the GPR gene signature was a risk factor for overall survival of BLCA patients and negatively correlated with the immune response of BLCA patients, while the TME signatures was a protective factor for overall survival of BLCA patients and positively correlated with the immune response of BLCA patients, and the interaction between the two signatures within each other needs to be further investigated. Second, when univariate Cox analysis was performed on immune cells to explore their effects with overall survival, we found that 12 immune cells were associated with overall survival of BLCA patients, but according to the current view of tumor immunology, the effects of some immune cells on overall tumor survival are not exact, and some immune cells have both pro-tumor and tumor suppressive effects, such as macrophages, and it is difficult for us to screen immune cells with protective effects according to KM curve and then conduct risk modeling. To ensure the reliability of this model, we followed the mainstream view of tumor immunology for modeling, but as tumor immunology continues to be studied in depth, this model may not be appropriate, and we are happy to adjust this model to make it more scientific and applicable. Finally, this gene signature lacks validation from large-scale perspective trials. In addition, the TCGA cohort consists of gene sequencing data, whereas GSE160693 is gene chip data, and data from external cohorts may not fully reveal the actual overall survival effect of this model in BLCA. Also, the detailed mechanism of action of this gene signature in BLCA has not been explored at the cellular and molecular level.

In conclusion, we propose a new GPR-related gene signature as a practical tool for BLCA patients, which better predicts the overall survival of BLCA patients and their response to immunotherapy.

## Supplementary Material

Supplementary material
