# Supplementary material for: The G protein-coupled receptor-related gene signatures for predicting prognosis and immunotherapy response in bladder urothelial carcinoma
Source: Open Life Sci. 2023 Aug 10;18(1):20220682. doi: 10.1515/biol-2022-0682 (PMC10426760; doi:10.1515/biol-2022-0682)
Supplement: Supplementary material [file biol-2022-0682-sm.pdf]

Supplementary material

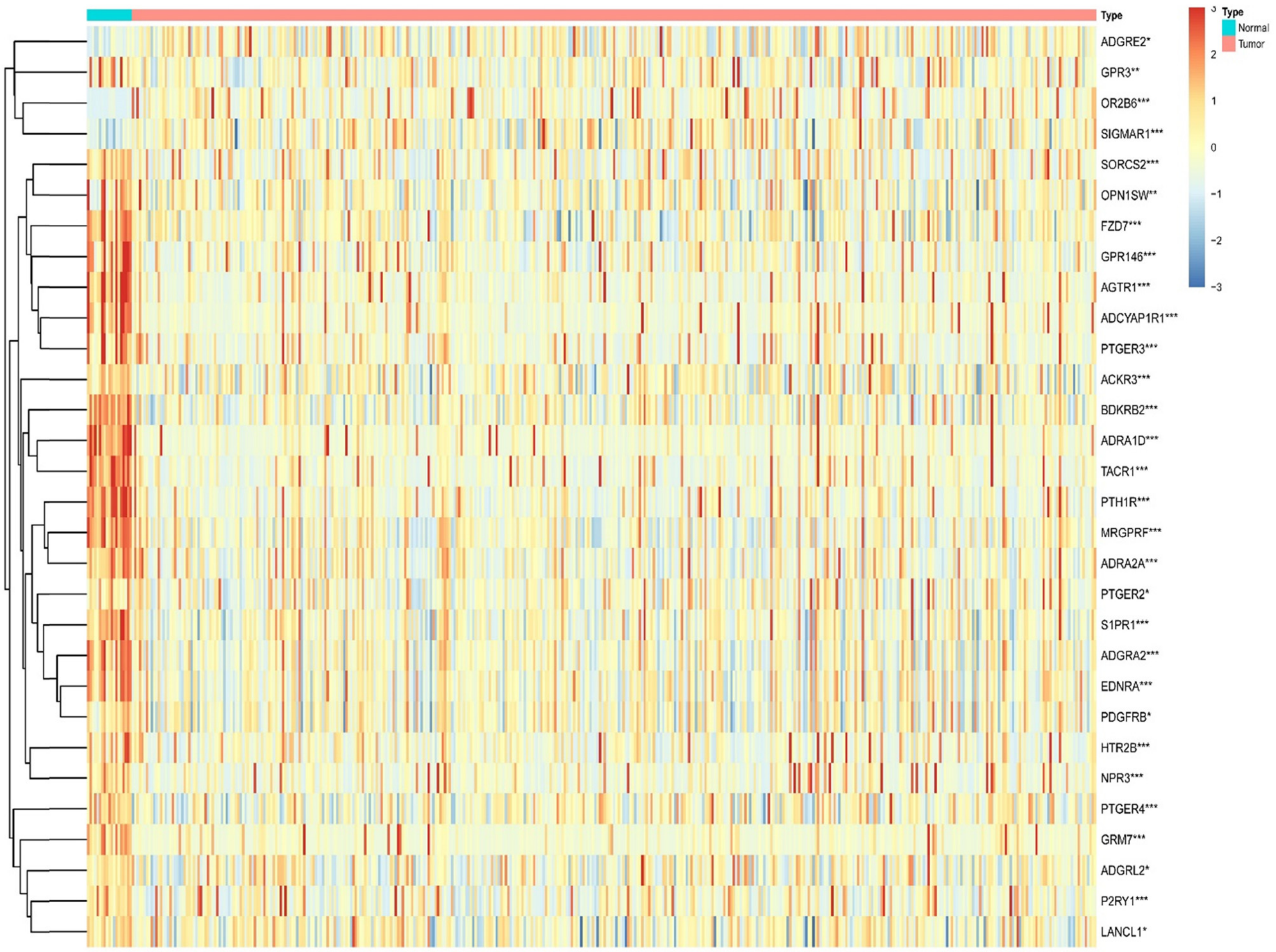

Figure S1: Heat map of differential expression of GPR-score gene signature in BLCA cancer tissues and normal tissues. **P* < 0.05; ***P* < 0.01; ****P* < 0.001.

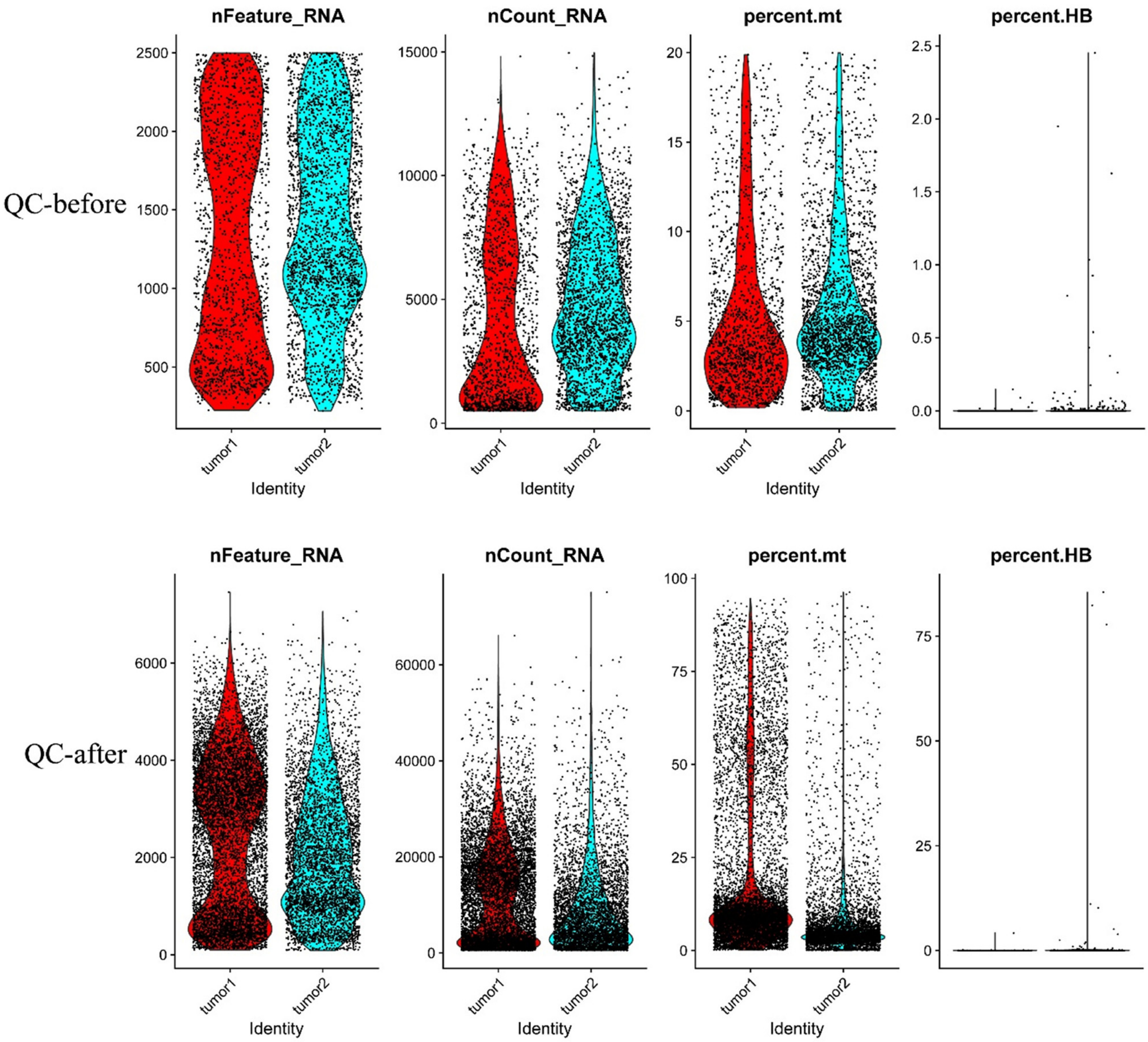

Figure S2: Cellular quality control violin plot of BLCA single cell transcriptome data. mt: mitochondrial gene; HB: hemoglobin gene.

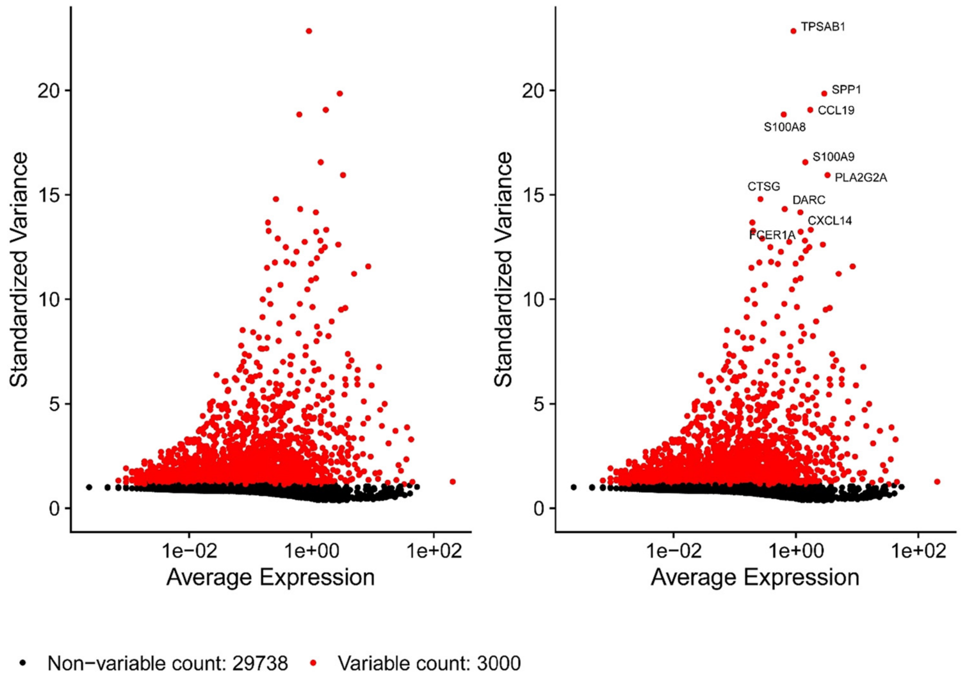

Figure S3: Scatter plot of the identification of highly variable genes in each subgroup after UMAP downscaling clustering.

Table S1: List of immune checkpoint-associated genes

BTN2A2 BTNL3 BTNL9 IDO1 TDO2 VTCN1 ADORA2A CD276 CD274
PDCD1LG2 PDCD1 CD80 CD86 CTLA4 CD160 BTLA TNFRSF14 HAVCR2
LGALS9 CD47 SIRPA TIGIT PVR BTN2A1 CD209 LAG3

Table S2: Molecular markers of cell types

**Main cell types**						
Epithelial cells	EPCAM	KRT19	PROM1	ALDH1A1	CD45+	CD24
Immune cells	PTPRC	MS4A1	JCHAIN	CD68	JCHAIN	MS4A1
Stomal cells	PECAM1	VWF	ACTA2	CD10+		
T cells	CD3D	CD3E	CD8A	CD4		
B cells	CD19	CD79A	MS4A1	CD20		
Endothelial cells	VWF	PECAM1				
Lymphatic cells	CCL21	PROX1				
Fibroblasts	PDGFRA	LUM	FGF7	MME		
Myofibroblasts	PDGFRA	LUM	MYLK	ACTA2	PDGFRB	
smooth muscle cells	MYLK	ACTA2	PDGFRB	MYH11		
Mesothelial cells	UPK3B	WT1				
Plasma cells	IGHG1	MZB1	SDC1	CD79A		
NK Cells	FGFBP2	CX3CR1	FCG3RA			
Monocytes, macrophages	CD68	CD163	CD14			
